# Elucidating Photochemical Conversion Mechanism of PDMS to Silica under Deep UV Light and Ozone

**DOI:** 10.1021/acs.jpclett.4c03477

**Published:** 2025-01-13

**Authors:** Harikrishna Sahu, Mingzhe Li, Madhubanti Mukherjee, Liang Yue, H. Jerry Qi, Rampi Ramprasad

**Affiliations:** ‡ School of Materials Science and Engineering, Georgia Institute of Technology, Atlanta, Georgia 30332, United States; ¶ The George W. Woodruff School of Mechanical Engineering, Georgia Institute of Technology, Atlanta, Georgia 30332, United States

## Abstract

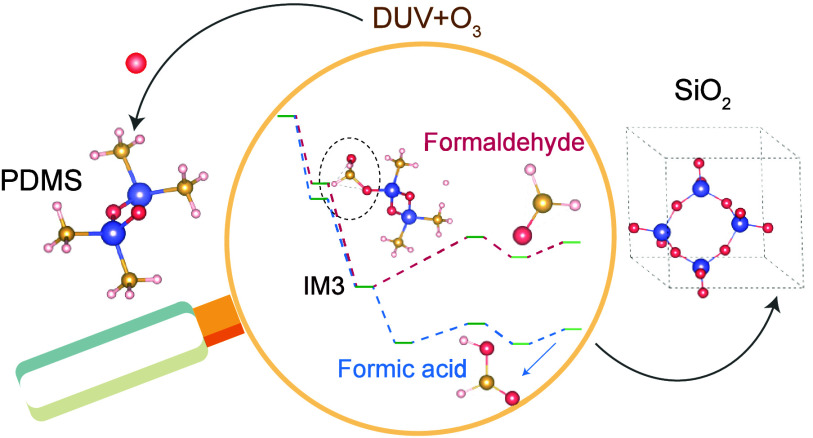

Photochemistry-based silica formation offers a pathway toward energy-efficient and controlled fabrication processes. While the transformation of poly(dimethylsiloxane) (PDMS) to silica (often referred to as SiO_*x*_ due to incomplete conversion) under deep ultraviolet (DUV) irradiation in the presence of oxygen/ozone has experimentally been validated, the detailed mechanism remains elusive. This study demonstrates the underlying molecular-level mechanism of PDMS-to-silica conversion using density functional theory (DFT) calculations. Our findings reveal that atomic oxygen plays a key role in converting PDMS to silica by catalyzing the replacement of -CH_3_ groups to -OH groups, with a barrier-less insertion into Si–C and C–H bonds, eventually leading to condensation reactions that produce silica and formaldehyde and/or formic acid as byproducts. The proposed molecular pathway has further been validated through controlled experiments, which confirm the successive -CH_3_ to -OH replacements and identify gaseous byproducts such as formaldehyde. These findings offer insights into the fundamental processes involved in photochemistry-based silica fabrication and could pave the way for advancements in energy-efficient materials synthesis.

Silica, or silicon dioxide (SiO_2_) is an important material with recognized attractive properties, including exceptional thermal stability, chemical inertness, and optical transparency, underpinning its multifaceted applications across diverse industrial domains and advanced engineering.^1−5^ Recent advances in three-dimensional (3D) printing technology have enabled the fabrication of intricate, precise, and microscale glass structures, opening new avenues for various fields including micro-optics, microfluidics, and lab-on-a-chip devices.^6,7^ However, the creation of silica structures through conventional 3D printing techniques generally requires the application of pyrolysis and sintering processes, necessitating high temperatures and extended durations,^6,8^ thereby presenting challenges and incurring substantial energy consumption. Oxygen plasma treatment, while widely used for surface modification, has well-known limitations: plasma-treated surfaces often experience rapid hydrophobic recovery within minutes of exposure, and prolonged treatment can lead to undesirable surface cracking.^9−12^ The integration of photochemistry heralds a paradigmatic shift toward more efficient, less demanding (e.g., low temperature) and controlled fabrication processes. Poly(dimethylsiloxane) (PDMS) and its derivatives have emerged as a promising precursor material for silica synthesis under deep ultraviolet (DUV) irradiation in the presence of oxygen or ozone.^13−16^ The tunability of the DUV+ozone-driven conversion of PDMS to silica allows for the precise adjustment of the resulting material properties, such as porosity, surface roughness, and chemical stability, making the process highly applicable in various fields, including flexible electronics, sensor technology, and biomedical devices. This photochemistry-based approach has been extensively studied for the surface modification of PDMS, typically resulting in modifications with thicknesses on the nanometer scale.^17−20^ Since the conversion depends on atomic oxygen penetration, which varies with depth, the chemical composition often deviates from pure silica and is commonly referred to as SiO_*x*_. Notably, in our recent work,^21^ we leveraged this approach to fabricate glass structures at the micrometer scale, achieving remarkable results with a maximum processing temperature of 220 °C (see ref [^21^] for detailed temperature profile) and a processing duration of merely 5 h.

Although this photochemistry-based 3D printing method has shown great promise, the mechanism underlying the conversion of PDMS to silica under DUV-ozone conditions remains speculative^14,15,22,23^ and has yet to be validated. This gap in understanding impedes advancements in technologies that aim to enhance the speed, resolution, and scalability of the 3D printing process. Specifically, the molecular-level conversion mechanism is not yet fully understood, which is crucial for designing new precursor polymers suitable for photochemistry-based ceramic synthesis. In our previous work,^21^ we performed density functional theory (DFT) simulations to investigate the roles of ozone, molecular oxygen, and atomic oxygen in the conversion of PDMS to silica. While DFT calculations suggested that the conversion is thermodynamically favorable, the detailed mechanism–particularly regarding the cleavage of Si–C bonds and the elimination of organic components–remained unclear. Additionally, further experimental observations showed that supplying only molecular oxygen or ozone did not result in the formation of silica. Similarly, we observed that treating PDMS with DUV radiation alone, in the absence of ozone, did not lead to significant conversion. These findings led us to conclude that atomic oxygen, which is generated in the absence of oxygen and thus ozone under DUV irradiation, is the key driving force behind the PDMS-to-silica conversion. Atomic oxygen is known for its high reactivity and is reported extensively in the literature for rapid addition or insertion reactions into target molecules to form various chemical species, including alcohols.^24−28^

Notably, the mechanisms of PDMS to silica conversion have been extensively investigated in the literature, including works that propose the formation of radical intermediates.^14,15,22,23^ For instance, the presence of radical species has been demonstrated under DUV treatment in the absence of atomic oxygen, while studies indicate that no significant electron paramagnetic resonance (EPR) signals are detected in the presence of oxygen.^23^ This observation suggests that the reaction mechanism may differ from those previously reported. Our work builds on these findings by focusing on the role of atomic oxygen generated from ozone under DUV irradiation. Unlike the radical mechanisms proposed earlier, our initial DFT calculations consistently indicate a barrierless insertion of atomic oxygen into C–H and Si–C bonds in the PDMS chain, rather than the hydrogen atom abstraction from the methyl group suggested in previous studies. However, it is noteworthy that DUV treatment may potentially induce radicals in PDMS, despite the absence of detectable EPR signals, and their possible interaction with atomic oxygen merits further investigation.

In this study, we propose a detailed molecular-level mechanism for the conversion of PDMS to silica in the presence of ozone and DUV light, utilizing density functional theory calculations and subsequently validated through experimental observations. The mechanism we developed arises from two key facts: the generation of atomic oxygens in a DUV-ozone environment and the rapid addition/insertion reactions involving atomic oxygen as reported in ref [^24−28^]. Figure 1 illustrates the schematic diagram of the proposed mechanism. The initial step in silica formation involves the replacement of -CH_3_ groups with -OH groups, catalyzed by highly reactive atomic oxygens generated upon exposure of O_2_/O_3_ molecules under DUV light. Atomic oxygens potentially get inserted into various Si–C and C–H bonds within the PDMS chain, leading to several possible intermediates, as illustrated in IM1-IM4. Subsequently, these intermediates undergo dissociation passes through transition states, leading to the replacement of a -CH_3_ group with an -OH group, forming PDMS–OH. The byproducts are determined by the intermediates formed during oxygen atom insertion into the PDMS chain. Employing a similar approach, all -CH_3_ groups are replaced, forming poly(dihydroxysiloxane) (PDHS). The PDHS chain interacts with neighboring PDHS chains and undergoes a series of condensation reactions to form interlinked structures. This process continues, ultimately leading to the formation of silica as the end product.

**Figure 1:**
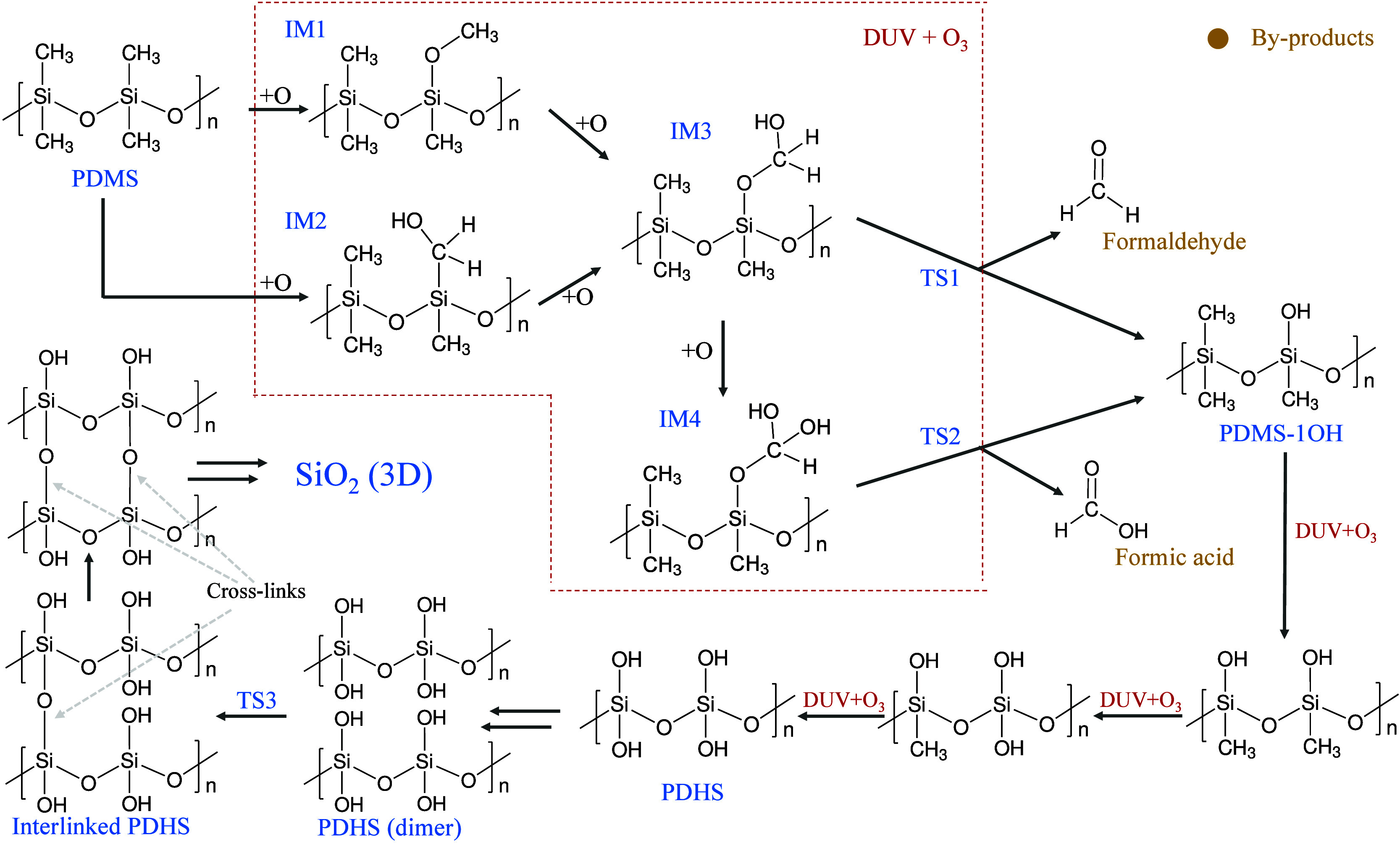
Schematic representation illustrating the poly(dimethylsiloxane) (PDMS) to silica conversion mechanism driven by ozone and deep UV, showcasing key molecular transitions.

To explore the mechanism at the molecular level, we conducted first-principles calculations employing the DFT framework, as implemented in the Vienna Ab Initio Simulation Package (VASP).^29,30^ In these calculations, we set a plane wave energy cutoff of 550 eV to accurately describe the electronic wave functions. The exchange-correlation (XC) energies were computed using the Perdew–Burke–Ernzerhof (PBE) functional. We ensured convergence in optimizing the structures when atomic forces dropped below 0.001 eV/Å. Additionally, for the optimization of transition state geometries, we employed the climbing image nudged elastic band (cNEB) method.^31,32^ For cNEB calculations, initially five intermediate images were considered between reactants and products, further increasing to 7 or 15 images when needed. Frequency calculations were carried out to confirm the absence of imaginary frequencies for ground state structures and the presence of one imaginary frequency for the transition state structures.

As a first step, we focused on the detailed investigation of replacing one -CH_3_ group with an -OH group, and the corresponding energy profile is presented in Figure 2(a). Given the abundance of atomic oxygen in DUV-ozone environment, it is highly likely that these reactive oxygen atoms interact with the PDMS chain. We approached the oxygen atoms from various possible directions toward the C–H and Si–C bonds of the PDMS chain. Notably, all scenarios demonstrated a barrier-less insertion of the oxygen atom, aligning with literature findings on analogous reactions.^26,27^

**Figure 2:**
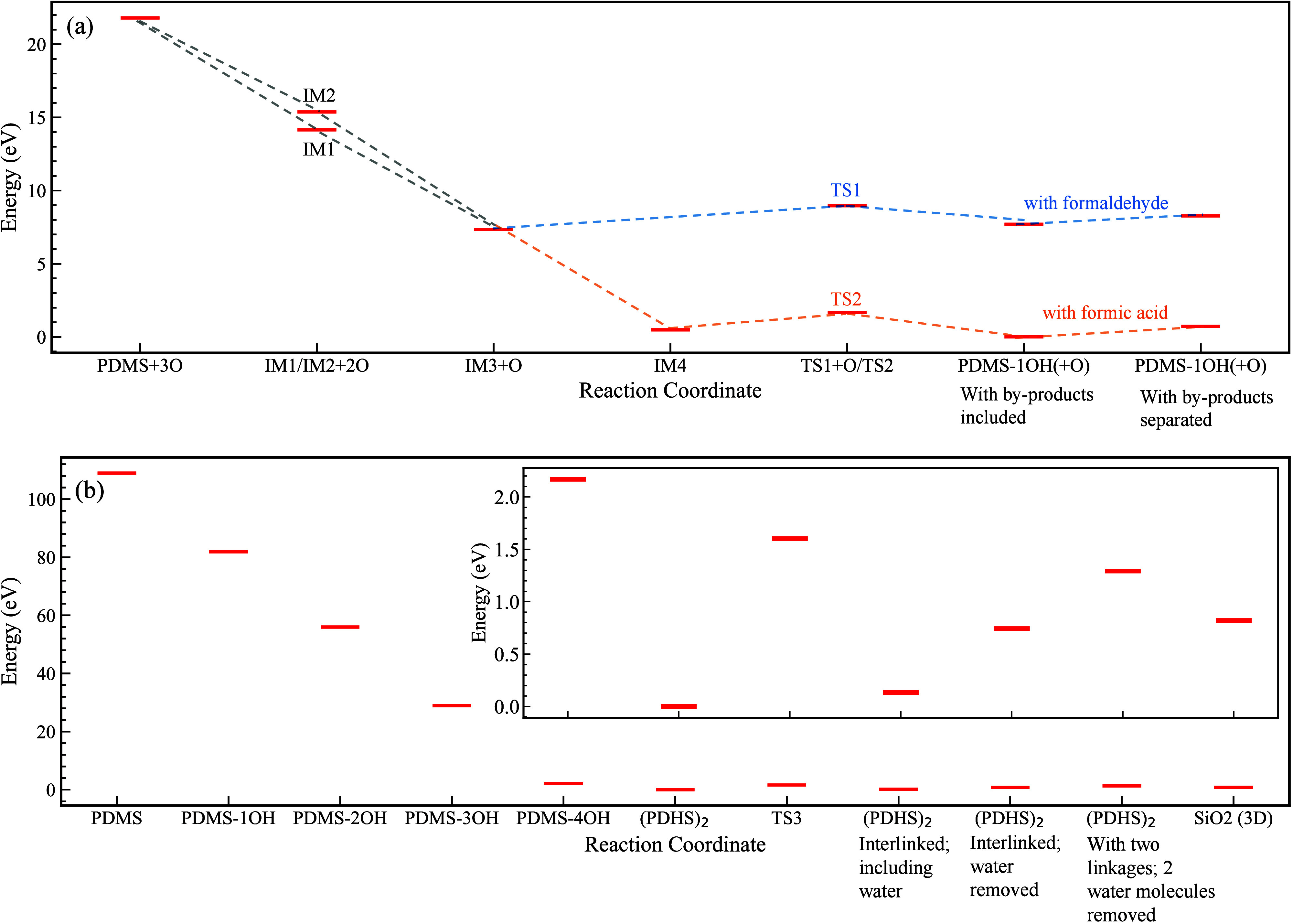
Potential energy diagram for poly(dimethylsiloxane) (PDMS) to silica conversion calculated using DFT. (a) Energy profile depicting the substitution of one -CH_3_ group in PDMS with an -OH group, resulting in PDMS–OH. (b) Overall energy profile for the complete PDMS to silica conversion process. Inset in (b) highlights the critical transition from poly(dihydroxysiloxane) (PDHS) to silica.

Intermediate IM3 was formed after the insertion of two oxygen atoms into Si–C and C–H bonds. IM3 proceeds through a transition state (TS1) and dissociates to PDMS–1OH and formaldehyde, encountering an energy barrier of 1.63 eV. The geometry of TS1 is depicted in Figure 3(a), where a hydrogen atom from an -OH group transfers to an oxygen atom bonded to a neighboring silicon atom, facilitating the removal of formaldehyde and subsequent attachment of an -OH group to a silicon atom.

**Figure 3:**
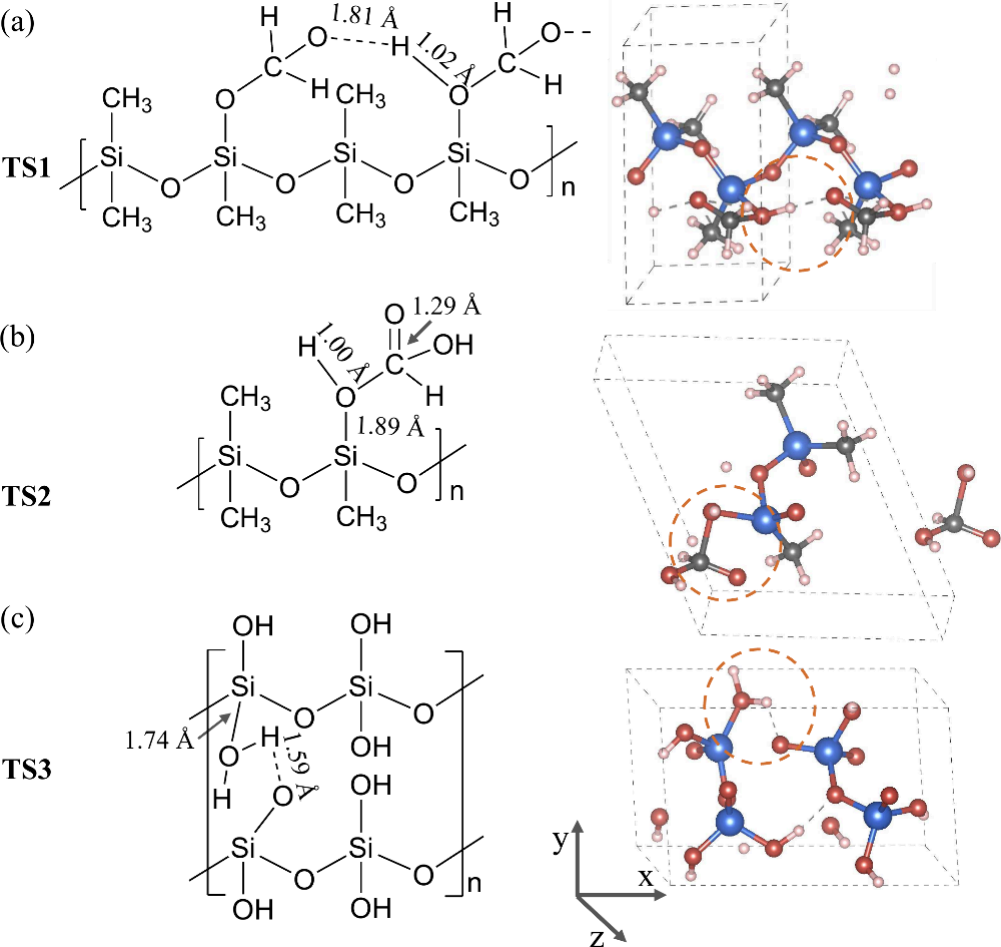
Representation of 2D and 3D geometries of three transition states involved in the poly(dimethylsiloxane) (PDMS) to silica conversion: (a) Transition State 1 (TS1), (b) Transition State 2 (TS2), and (c) Transition State 3 (TS3).

**Figure 4:**
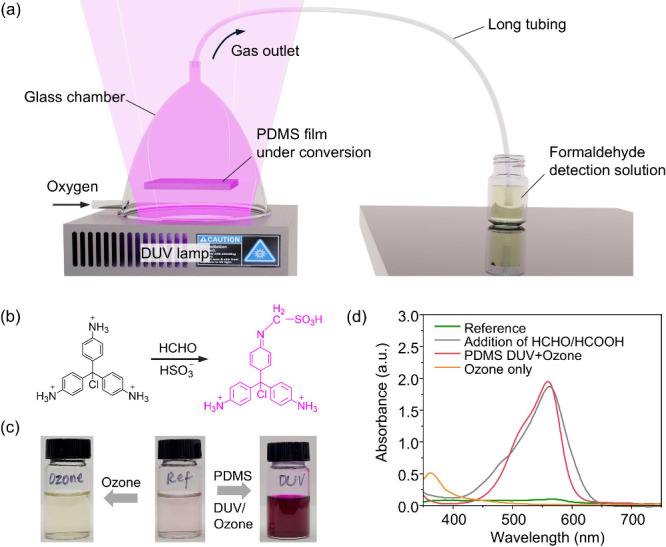
Detection of formaldehyde, a byproduct generated in our proposed mechanism. (a) Schematic of the experiment setup. (b) The formaldehyde detection mechanism in the modified pararosaniline method. (c and d) The photo images and UV–vis spectra of detection solutions.

Given the ample availability of oxygen atoms, there is a possibility of forming IM4 by inserting another oxygen atom into the C–H bond. Similar to IM3, IM4 undergoes transition state TS2 with an energy barrier of 1.12 eV to form PDMS–1OH and formic acid as the byproduct. In the TS2 structure in Figure 3(b), akin to the previous transition state, a hydrogen atom is transferred from an -OH group to an oxygen atom attached to a silicon atom, facilitating the removal of formic acid, followed by the formation of PDMS–1OH. It is noteworthy that the small energy barriers for these paths contribute to the efficient replacement of the methyl group with hydroxyl groups.

Considering the analogous process for replacing other -CH_3_ groups with -OH groups, we investigated the complete conversion of PDMS to silica, with the corresponding energy profile depicted in Figure 2(b). Notably, the successive replacement of -CH_3_ groups with -OH groups is thermodynamically favorable. Upon obtaining PDHS, we arranged two chains together in a unit cell to study the interaction between them. As anticipated, a condensation reaction between two adjacent -OH groups was observed, leading to the formation of interlinked PDHS, accompanied by the subsequent removal of a water molecule. This step passes through a transition state (TS3) with an energy barrier of 1.60 eV. The structure of TS3, depicted in Figure 3(c), indicates the transfer of an H atom from one -OH group to a neighboring -OH group attached to a different Si-atom, followed by the elongation of the neighboring Si–O bond. Ultimately, a water molecule is removed, and a new bond between the neighboring silicon atom and the oxygen atom is formed. While DFT calculations indicate relatively high energy barriers for the conversion process, it is important to note that the system temperature rises to approximately 220 °C during DUV irradiation, even without external heating. This elevated temperature plays a crucial role in overcoming the energy barriers, thus facilitating the reaction. The combination of heat generated by DUV exposure and the presence of reactive atomic oxygen ensures the progress of the PDMS-to-silica conversion.

Additionally, we observed that the removal of each water molecule from the system requires a certain energy (∼0.60 eV), as water molecules typically form hydrogen bonds with oxygen atoms in the PDHS system. Despite this energetic cost, the final product, silica, which forms after the removal of four water molecules in our study, exhibits notable stability. This contributes significantly to driving the reaction toward the desired end product, highlighting the thermodynamic feasibility of the process.

The proposed molecular-level mechanism (see Figure 1) demonstrates two possible byproducts, formed during the conversion of PDMS to PDHS: formaldehyde and formic acid. Given the presence of UV–visible radiation and strong oxidizing agents in the reaction environment, such as ozone and atomic oxygen, there is a strong possibility that formaldehyde oxidizes to formic acid.^33−35^ This imposes a challenge to distinguish whether the formic acid resulted from the conversion of PDMS to silica or from the oxidation of formaldehyde. Therefore, to validate our proposed mechanism, we conducted tests to detect formaldehyde alone. Figure 4(a) illustrates the experimental setup. A xenon lamp (LH-810, XENON Corp., Wilmington, MA, USA) was used as the light source, which emits high-intensity DUV light. A thin film of PDMS (50 μm thickness) was positioned 5 mm away from the light source within a customized glass chamber. This chamber was continuously purged with purified oxygen to facilitate the conversion of PDMS to silica. The resultant gaseous phase was then directed through a 3-m long tubing and captured in a detection solution. The long tubing ensures that all short-lived species, such as atomic oxygen, are eliminated, leaving only stable gas products in the gaseous phase for analysis. We then employed a modified pararosaniline method^36^ to detect formaldehyde, a byproduct in our proposed mechanism. The detection solution consists of 10 mL of 0.52 mM pararosaniline in a 0.24 M HCl aqueous solution. After bubbling the gaseous phase from the PDMS conversion process into this solution for 30 min, we added 1 mL of 8 mM sodium sulfite aqueous solution and mixed thoroughly. The solution was then maintained at 25 °C for 1 h to stabilize the color. Figure 4(b) illustrates the detection mechanism: acidified pararosaniline reacts with formaldehyde and sulfur dioxide to form a magenta-colored alkylsulfonic acid chromophore.

The photo images and UV–vis spectra measured using a UV–vis-NIR spectrometer (AvaSpec-ULS2048CL-EVO, Avantes B.V., Apeldoorn, Netherlands) of the detection solutions are displayed in Figure 4(c) and 4(d). A detection solution without the gaseous phase served as the reference, which showed a light color with a broad but low absorption band from 350 to 650 nm. In contrast, the solution postgaseous phase introduction exhibits a dark magenta color with a characteristic absorption peak at 565 nm, indicating the presence of formaldehyde. This is also consistent with the UV–vis spectra of detection solution with the addition of pure formaldehyde and formic acid. To eliminate the effect of ozone, we conducted a control experiment where only ozone was bubbled into the detection solution using an ozone generator (VMUS-4, Oxidation Technologies, LLC., Inwood, IA, USA). The resultant solution exhibited a light yellow color with an absorption peak at 360 nm, indicative of the oxidation of pararosaniline by ozone, aligning with previous findings.^37^ These observations confirm that formaldehyde was generated during the substitution of -CH_3_ groups in PDMS with -OH groups under DUV-ozone condition, corroborating our proposed molecular-level photochemistry mechanism as depicted in Figure 2(a).

To further validate the proposed PDMS to silica conversion pathway, we employed Fourier-transform infrared spectroscopy (FTIR, ATR mode, Nicolet iS5, Thermo Fisher Scientific Inc., Waltham, MA, USA) to characterize the PDMS thin film after undergoing conversion for different durations. Figure 5(a) presents the FTIR absorbance spectra across the range of 2600 to 3800 cm^–1^. Upon DUV-ozone treatment, the O–H band centered at 3350 cm^–1^ showed a progressive increase,^15^ reaching a maximum at 1 h as depicted in Figure 5(b). This increase indicates the generation of -OH groups and the formation of PDHS. Meanwhile, the characteristic peaks at 2961 and 2905 cm^–1^, corresponding to the symmetric and asymmetric stretching of the C–H bonds, is reduced due to the removal of methyl groups, as elucidated in Figure 1. Further DUV irradiation to 3 h results in the near-complete elimination of this broad band, revealing the condensation reaction of neighboring -OH groups. The PDMS to silica conversion pathway was also experimentally validated using solid-state nuclear magnetic resonance (NMR) spectroscopy (Avance 400 III HD, Bruker, Billerica, MA, USA). Figure S1 presents the ^1^H and ^29^Si MAS NMR spectra of the converted sample over various durations. The emergence and shifts of characteristic peaks within these spectra clearly demonstrate the substitution of -CH_3_ groups with -OH groups, followed by condensation to SiO_2_, corroborating the FTIR findings. Therefore, the proposed mechanism for the conversion of PDMS to PDHS and its ultimate condensation to SiO_2_, is consistent with the experimental measurements. By characterizing the carbon atomic ratio on the cross-section of the film, we confirmed that the conversion process is nearly complete after 3 h of DUV irradiation (Figure S2). This finding is crucial for applications such as the 3D printing of silica microstructures, where complete conversion ensures the integrity and functionality of the printed objects.

**Figure 5:**
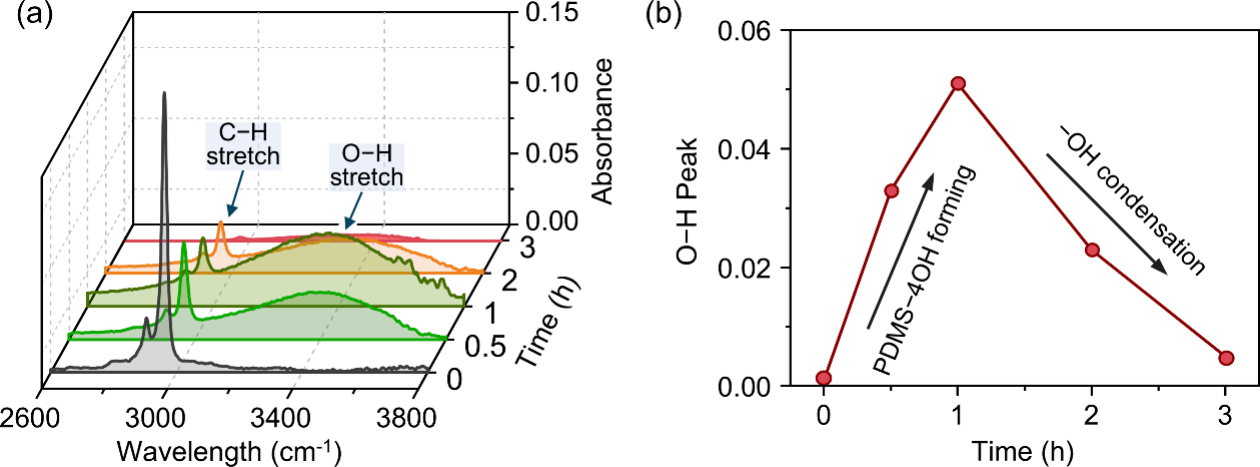
Experimental validation of the proposed conversion pathway from PDMS to PDHS and ultimately to SiO_2_. (a) FTIR spectra of the film sample after conversion for various durations. (b) Absorbance peak of the -OH group of the film samples at various conversion time.

In conclusion, atomic oxygen under DUV-ozone conditions plays a pivotal role in driving the conversion of PDMS to silica. This process commences with a barrier-less insertion of atomic oxygens into Si–C and C–H bonds, generating different intermediates. These intermediates pass through transition states with energy barriers less than 1.7 eV to replace -CH_3_ groups with -OH groups and subsequent condensation reactions lead to the formation of silica. The proposed molecular mechanism has further been supported by controlled experiments, which identify byproducts generated during the PDMS to silica conversion. By elucidating these molecular pathways and byproducts, this study not only enhances our understanding of the PDMS-to-silica conversion process but also lays the groundwork for future investigations into depth-dependent oxidation profiles and glassy-skin formation. Such insights could pave the way for advancements in photochemistry-based facile low-temperature 3D printing technologies for polymer-derived ceramics.

## Supporting Information Available

Characterization data, including solid-state MAS NMR spectra (^1^H and ^29^Si) validating the PDMS-to-SiO_2_ conversion pathway, and quantitative elemental analysis via XPS for silica derived from PDMS films (PDF)
