# Supplementary material for: Elucidating Photochemical Conversion Mechanism of PDMS to Silica under Deep UV Light and Ozone
Source: J Phys Chem Lett. 2025 Jan 13;16(3):747–53. doi: 10.1021/acs.jpclett.4c03477 (PMC11770750; doi:10.1021/acs.jpclett.4c03477)
Supplement: Supplementary file 1 — jz4c03477_si_001.pdf [file jz4c03477_si_001.pdf]

Supporting Information: Elucidating Photochemical Conversion Mechanism of PDMS to Silica under Deep UV Light and Ozone

Harikrishna Sahu, [*,†,¶] Mingzhe Li,[‡] Madhubanti Mukherjee,[†] Liang Yue,[‡] H. Jerry Qi,[‡] and Rampi Ramprasad[*,†]

† *School of Materials Science and Engineering, Georgia Institute of Technology, Atlanta, GA 30332, USA*

‡ *The George W. Woodruff School of Mechanical Engineering, Georgia Institute of Technology, Atlanta, GA 30332, USA*

¶*These authors contributed equally to this work.*

E-mail: hsahu3@gatech.edu; rampi.ramprasad@mse.gatech.edu

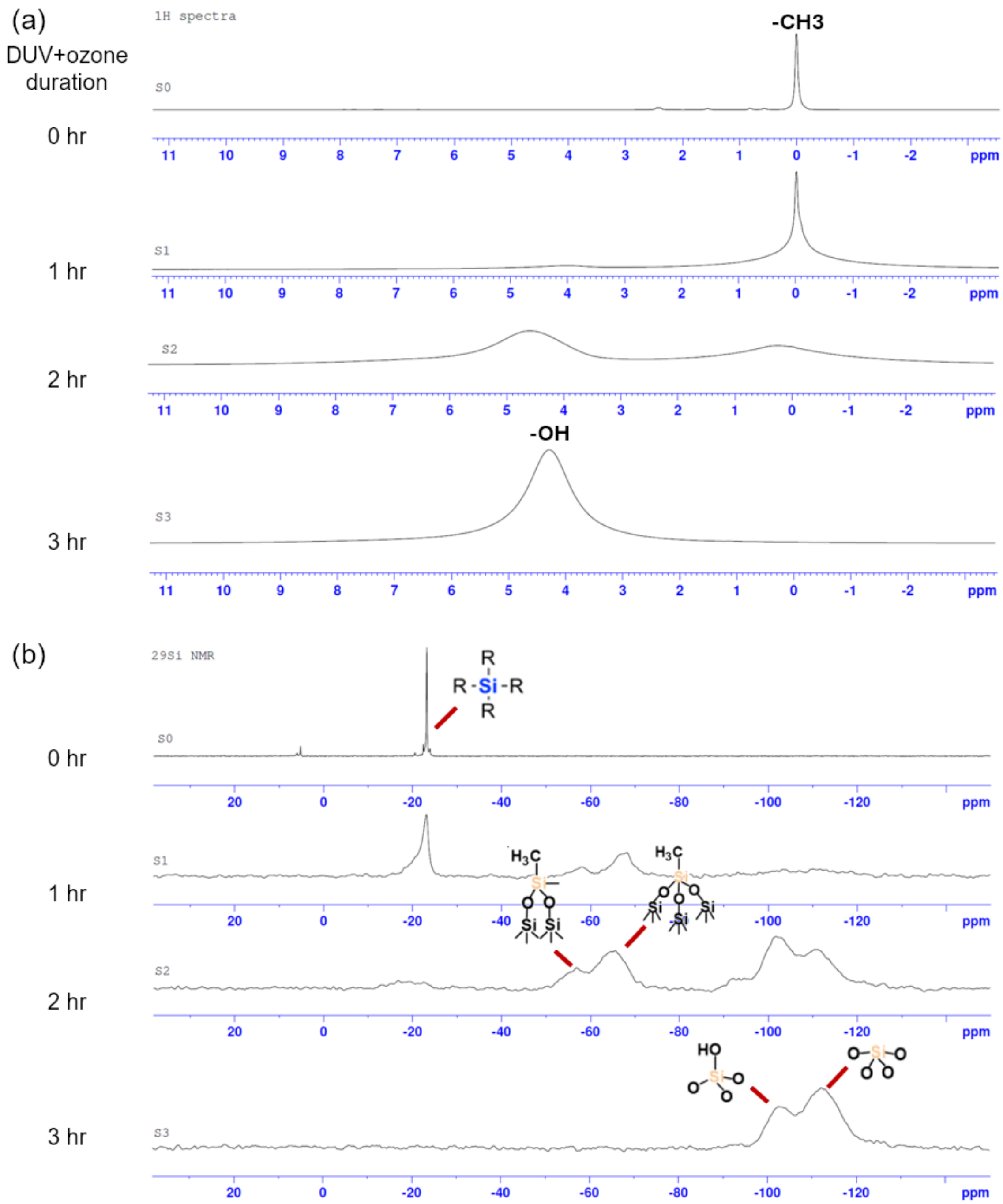

Figure S1. Validation of the proposed conversion pathway from PDMS to PDHS and ultimately to SiO_2. (a) Solid-state ^1H MAS NMR spectra recorded on a Bruker Avance 400 III HD spectrometer (Bruker, USA) at resonance frequency of 10 kHz. As the DUV+ozone treatment progresses, the methyl groups are substituted by hydroxyl groups. (b) Solid-state ^{29}Si MAS NMR spectra. As the DUV+ozone treatment progresses, the oxygen atoms are continuously inserted in between the -R-Si-R- groups, finally leading to the formation of SiO_2. The spectra shifts are consistent with ref [1] and our FTIR results, which supports the DFT simulation results experimentally.

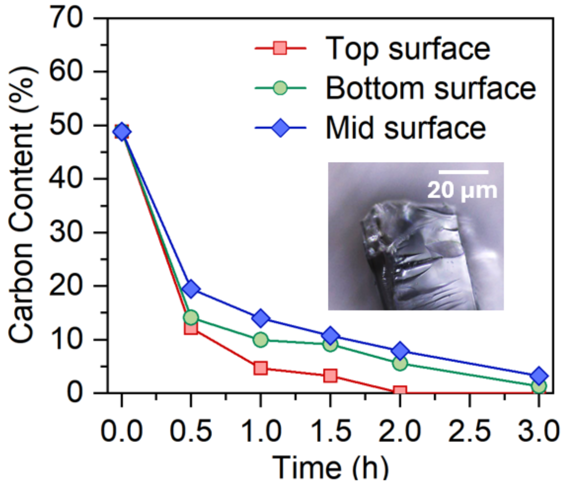

Figure S2. The carbon content on the top surface, the bottom surface, and the mid plane of the silica converted from 50 μm thick PDMS film. Quantitative elemental analysis was performed using an XPS (K-Alpha, Thermo Fisher Scientific Inc., Waltham, MA, USA). Inset shows the optical image of the cross-section of the film after 3-hour DUV+ozone treatment.

References

(1) Protsak, I. S.; Morozov, Y. M.; Dong, W.; Le, Z.; Zhang, D.; Henderson, I. M. A ^{29}Si, ^{1}H, and ^{13}C solid-state NMR study on the surface species of various depolymerized organosiloxanes at silica surface. *Nanoscale Res. Lett.*, **2019**, 14, 160.
